# Mechanism of action of icaritin on uterine corpus endometrial carcinoma based on network pharmacology and experimental evaluation

**DOI:** 10.3389/fonc.2023.1205604

**Published:** 2023-07-19

**Authors:** Yan-Bin Jin, Xiao-Chen Liang, Jun-Hong Cai, Kang Wang, Chen-Yang Wang, Wen-Hua Wang, Xiu-Li Chen, Shan Bao

**Affiliations:** ^1^ Department of Gynecology and Obstetrics, Hainan Affiliated Hospital of Hainan Medical University, Hainan General Hospital, Haikou, Hainan, China; ^2^ Key Laboratory of Reproductive Health Diseases Research and Translation (Hainan Medical University), Ministry of Education, Haikou, Hainan, China; ^3^ Hainan Provincial Key Laboratory for Human Reproductive Medicine and Genetic Research, The First Affiliated Hospital of Hainan Medical University, Hainan Medical University, Haikou, Hainan, China; ^4^ Medical Laboratory Center, Hainan Affiliated Hospital of Hainan Medical University, Hainan General Hospital, Haikou, Hainan, China; ^5^ Department of Obstetrics and Gynecology, The Second Affiliated Hospital of Zhengzhou University, Zhengzhou, Henan, China; ^6^ Department of Obstetrics and Gynecology, The First Hospital of Lanzhou University, Lanzhou, Gansu, China

**Keywords:** uterine corpus endometrial carcinoma (UCEC), icaritin, network pharmacology, PI3K, Akt

## Abstract

**Background:**

Uterine corpus endometrial carcinoma (UCEC) belongs to a group of epithelial malignant tumors. Icaritin is the main active compound of Epimedii Folium. Icaritin has been utilized to induce UCEC cells to death.

**Methods:**

We wished to identify potential targets for icaritin in the treatment of UCEC, as well as to provide a groundwork for future studies into its pharmacologic mechanism of action. Network pharmacology was employed to conduct investigations on icaritin. Target proteins were chosen from the components of icaritin for UCEC treatment. A protein–protein interaction (PPI) network was established using overlapping genes. Analyses of enrichment of function and signaling pathways were undertaken using the Gene Ontology (GO) and Kyoto Encyclopedia of Genes and Genomes (KEGG) databases, respectively, to select “hub genes”. Finally, experiments were carried out to ascertain the effect of icaritin on endometrial cancer (HEC-1-A) cells.

**Results:**

We demonstrated that icaritin has bioactive components and putative targets that are therapeutically important. Icaritin treatment induced sustained activation of the phosphoinositide 3-kinase/protein kinase B (PI3K/Akt pathway) and inhibited growth of HEC-1-A cells.

**Conclusion:**

Our data provide a *rationale* for preclinical and clinical evaluations of icaritin for UCEC therapy.

## Introduction

Uterine corpus endometrial carcinoma (UCEC) belongs to a group of epithelial malignant tumors. It is the most common gynecologic malignancy in developed countries, and occurs in the endometrium of primarily postmenopausal and perimenopausal women (1).

The morbidity and mortality of UCEC has shown an increasing trend worldwide in recent years (2). Surgery combined with adjuvant therapy (radiotherapy and/or chemotherapy) is the standard treatment for UCEC (3). Although efficacious in a subset of patients, these treatments can elicit severe side-effects. In clinical treatments of cancer, prolonged chemotherapies can induce drug resistance that leads to treatment failure (4).

Icaritin is the main active compound of Epimedii Folium (5). Icaritin has anti-tumor activity and can also be used with other anti-tumor agents (e.g., curcumin, doxorubicin) to treat tumors (6). It has been reported that icaritin can inhibit the growth of endometrial cancer cells (7). Few reports have focused on the mechanism of action of icaritin. The precise molecular mechanisms of its anti-tumor effect have not been characterized.

We aimed to provide a reference for UCEC cells death mechanistic research. Using network pharmacology, we investigated how icaritin may be able to regulate UCEC. We used a compounds–genes–disease (C–G–D) network to create a “core” between icaritin and UCEC. We identified overlapping genes to establish a protein–protein interaction (PPI) network. Next, we perused the Gene Ontology (GO) and Kyoto Encyclopedia of Genes and Genomes (KEGG) databases to analyze the enrichment of function and signaling pathways, respectively, to select “hub genes.” Finally, to ascertain how icaritin affects cell growth and the phosphoinositide 3-kinase/protein kinase B (PI3K/Akt pathway), *in vitro* cellular experiments were undertaken.

## Materials and methods

### Bioactive ingredients of icaritin

To identify all of the metabolites present in icaritin, we utilized the Traditional Chinese Medicine Systems Pharmacology (TCMSP) database (https://tcmsp-e.com/). Oral bioavailability (OB) and “drug-likeness” (DL) are the essential pharmacokinetic qualities in absorption, distribution, metabolism, and excretion methodology. OB refers to the pace and extent of absorption of an oral medication into the circulatory system from the gastrointestinal tract. DL refers to herbal compounds that are related structurally to recognized medicines. Substances with OB ≥30% and DL ≥0.18 were investigated as active ingredients in the present study (8).

### Products of icaritin genes are activated

By attaching to certain molecular processes at transcriptome and protein levels, chemicals can have an impact upon biosynthesis. The TCMSP database was used to find chemical–target interactions. The “Ensemble” dataset within the UniProt database (www.uniprot.org/) was used to harmonize gene names. After deleting duplicate targets, the targets of icaritin were identified.

### Collection of disease targets

We used the phrase “UCEC” to search the GeneCards database (www.genecards.org/) and the Online Mendelian Inheritance in Man (OMIM) database (www.omim.org/) and Therapeutic Target Database (https://db.idrblab.net/) to search for UCEC-related targets. The GeneCards database includes complete data about annotated human genes. Using the program “Venny 2.1.0”, the overlap between the putative target genes of icaritin and UCEC-related targets was found (http://bioinfogp.cnb.csic.ed/tools/venny/). These were considered to be targets in icaritin for UCEC intervention, as shown in a Venn diagram.

### Building of a C–G–D network

We used the target genes of icaritin and the treatment targets of UCEC from Venny 2.1.0, which was the overlap between them. Then, using Cytoscape 3.8.0 (https://cytoscape.org/), we merged the details from the ingredient, genetic, and disease data to create a C–G–D network (9).

### Analyses of a PPI network

To locate the genes that overlapped, we utilized the target genes of icaritin and the therapy targets of UCEC from either of the three datasets. The created PPI network, which itself was based on the Search Tool for the Retrieval of Interacting Genes/Proteins (STRING) database (https://string-db.org/), was displayed in Cytoscape. With a level of precision, the “degree” within Cytoscape was used to identify the top-11 domains that were plausible hub candidates (10).

### Enrichment analyses

Enrichment analyses were employed to provide further information about the biological activities of icaritin that occur in UCEC. We used the Metascape database (https://metascape.org/) for 135 key target genes. Then, in the GO database, biological process (BP), cellular component (CC), and molecular function (MF) terms were used to identify the top-10 GO keywords to obtain gene functions. The KEGG database was employed to identify the signaling pathways that were enriched. The Bioinformatics databases (www.bioinformatics.com.cn/) was used to conduct mapping analysis.

### Preparation of icaritin

Icaritin (purity = 99.27%) was purchased from MedChemExpress (catalog number: HY-N0678; Monmouth Junction, NJ, USA). Icaritin powder was dissolved in dimethyl sulfoxide to a high concentration (10 mM) and stored in the dark at −20°C. At the time of use, icaritin solutions were diluted with culture medium to the required concentrations.

### Culture and treatment of cells

The HEC-1-A cell line was obtained from ProCell (Santa Ana, CA, USA). The cell line was cultured in Dulbecco’s modified Eagle’s medium supplemented with 10% fetal bovine serum, 1% penicillin–streptomycin solution, 1% alanyl-glutamine in an atmosphere of 5% CO_2_ at 37°C.

### Cell-proliferation assays

During the logarithmic growth phase, HEC-1-A cells were plated into 96-well plates (1×10^4^ cells in a suspension of 100 μL), with three replicates per treatment. When cells showed adherent growth, the supernatant was replaced with medium containing icaritin. After 24h,48 h,72h 10 μL of Cell Counting Kit-8 (CCK-8) working solution (Servicebio, Wuhan, China) was added, and cells were cultured for an additional 2 h. The absorbance at 450 nm was measured. Cell-growth curves were drawn based on the average absorbance of each sample.

### Colony-formation assay

HEC-1-A cells were seeded in 12-well plates at 250 cells/well. When cells showed adherent growth, medium containing icaritin was added, followed by incubation at 37°C for 2 weeks, during which time the medium was changed every 3 days. Colonies were fixed with methanol, stained with 0.1% crystal violet, and counted.

### Wound-healing assay

Straight lines at a distance of 0.5 cm from each other were marked on the underside of six-well plates. Logarithmic-growth phase cells were seeded into dishes. Nicks were made 0.5-cm apart on the inside bottom of the dish along with the labeled lines using a 1000 μL sterile pipette tip when cells were close to confluence. Then, cell cultures were washed thrice with sterile phosphate-buffered saline and incubation in serum-free medium for 24 h was undertaken. The scratch width was photographed under phase contrast on an inverted microscope (BX51; Olympus, Tokyo) with a digital camera (100× magnification; DP70; Olympus). This experiment was repeated three times.

### Detection of apoptosis

Cells were seeded into six-well plates and cultured for 48 h. Flow cytometry was used to detect apoptosis in different groups according to manufacturer (BD Biosciences, San Jose, CA, USA) instructions. This experiment was repeated three times.

### Western blotting

Proteins extracted from HEC-1-A cells were prepared using RIPA buffer containing the phosphatase inhibitor phenylmethylsulfonyl fluoride (RIPA:phenylmethylsulfonyl fluoride = 100:1). Proteins were quantified with a bicinchoninic acid protein assay kit (Servicebio, Wuhan, China). Then, protein samples were fractionated by sodium dodecyl sulfate–polyacrylamide gel electrophoresis on 10% gels and transferred to polyvinylidene difluoride (PVDF) membranes. PVDF membranes were incubated with primary antibodies against PI3K (1:500 dilution; Proteintech, Chicago, IL, USA), phosphorylated (p)-PI3K (1:1000; BIOSS, Woburn, MA, USA), Akt (1:20000; Proteintech), p-Akt (1:20000; Proteintech), and glyceraldehyde 3-phosphate dehydrogenase (1: 50000; Proteintech). After washing with Tris-buffered saline and Tween 20, PVDF membranes were incubated with the corresponding secondary antibody for 1.5 h at 25°C. An electrochemiluminescence detection reagent (Beyotime Institute of Biotechnology, Shanghai, China) was used to visualize protein bands.

### Statistical analyses

Experimental data were analyzed using SPSS 24.0 (IBM, Armonk, NY, USA). Repeated-measures analysis of variance was used to compare differences among groups. The unpaired Student’s *t-*test was employed to compare mean values between two groups. Univariate analysis of variance was used to compare data between groups. Data are the mean ± standard deviation. *P* < 0.05 was considered significant.

## Results

### Active compounds present in icaritin

We searched the TCMSP database for 23 ingredients based on OB and DL values (**Table 1**).

**Table 1:** The 23 substances found in icaritin, along with their OB and DL values.

No	MoI ID	Molecule name	OB%	DL
1	MOL001510	24-epicampesterol	37.58	0.71
2	MOL001645	Linoleyl acetate	42.10	0.20
3	MOL001771	Poriferast-5-en-3beta-ol	36.91	0.75
4	MOL001792	DFV	32.76	0.18
5	MOL003044	Chryseriol	35.85	0.27
6	MOL003542	8-Isopentenyl-kaempferol	38.04	0.39
7	MOL000359	Sitosterol	36.91	0.75
8	MOL000422	Kaempferol	41.88	0.24
9	MOL004367	Olivil	62.23	0.41
10	MOL004373	AnhydroIcaritin	45.41	0.44
11	MOL004380	C-Homoerythrinan,1,6-didehydro-3,15,16-trimethoxy-, (3.beta)-	39.14	0.49
12	MOL004382	Yinyanghuo A	56.96	0.77
13	MOL004384	Yinyanghuo C	45.67	0.5
14	MOL004386	Yinyanghuo E	51.63	0.55
15	MOL004388	6-hydroxy-11,12-dimethoxy-2,2-dimethyl-1,8-dioxo-2,3,4,8-tetrahydro-1H-isochromeno[3,4-h] isoquinolin-2-ium	60.64	0.66
16	MOL004391	8-(3-methylbut-2-enyl)-2-phenyl-chromone	48.54	0.25
17	MOL004394	AnhydroIcaritin-3-O-alpha-L-rhamnoside	41.58	0.61
18	MOL004396	1,2-bis(4-hydroxy-3-methoxyphenyl) propan-1,3-diol	52.31	0.22
19	MOL004425	Icariin	41.58	0.61
20	MOL004427	Icariside A7	31.91	0.86
21	MOL000006	Luteolin	36.16	0.25
22	MOL000622	Magnograndiolide	63.71	0.19
23	MOL000098	Quercetin	46.43	0.28

### Probable targets of icaritin for UCEC therapy

**Figure 1:**
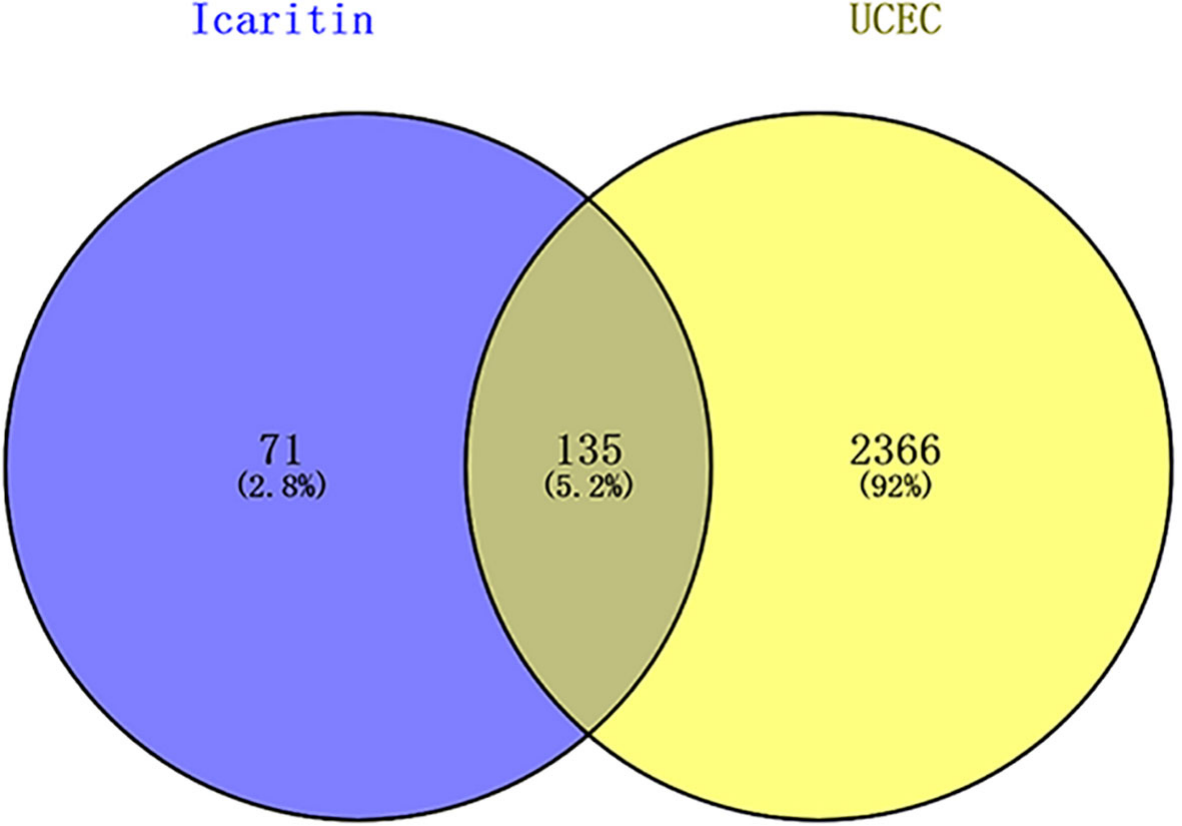
Venn diagrams revealed 135 candidate genes related to UCEC and icaritin.

After deleting duplicates, according to TCMSP and UniProt databases, icaritin had 206 targets. After repetitions in the GeneCards database had been identified, 2551 genes related to UCEC were identified. The intersection of 206 icaritin endpoints and 2551 UCEC-related keywords was established using Venny 2.1.0. Venn diagrams revealed 135 candidate genes related to UCEC and icaritin (**Figure 1**).

### Icaritin−related network

**Figure 2:**
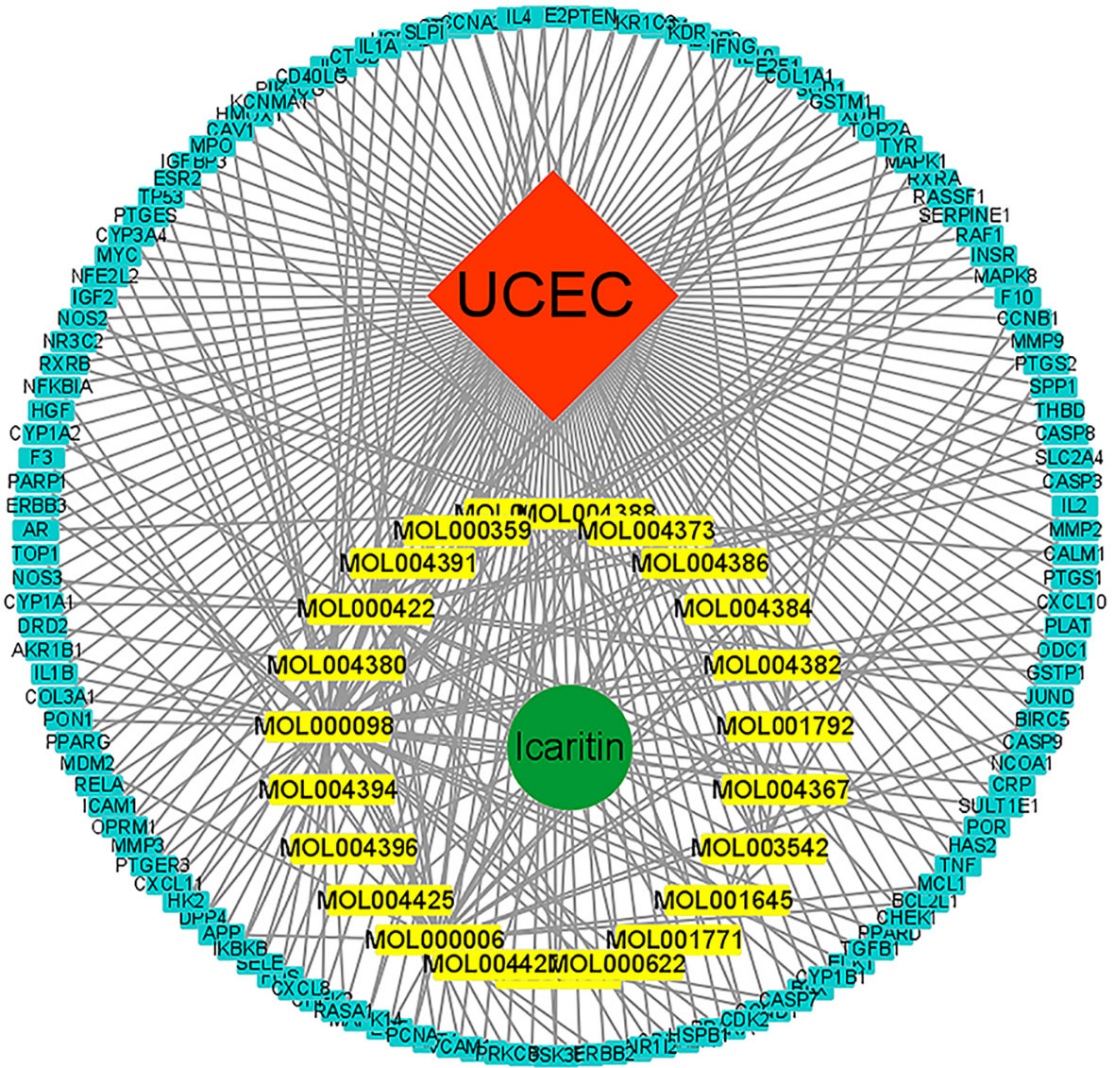
C–G–D network for icaritin. Molecules are represented in the inner circle. The gene of interest is in the outer circle.

The components of traditional Chinese medicine (TCM) formulations interact with a vast array of moieties to create a multitude of therapeutic actions. However, network pharmacology is an essential method for comprehending the basic mechanisms of how TCM formulations work. Cytoscape was used to create a C–G–D system for icaritin with regard to UCEC (**Figure 2**). The C–G–D system revealed 160 nodes and 293 edges.

### PPI network

**Figure 3:**
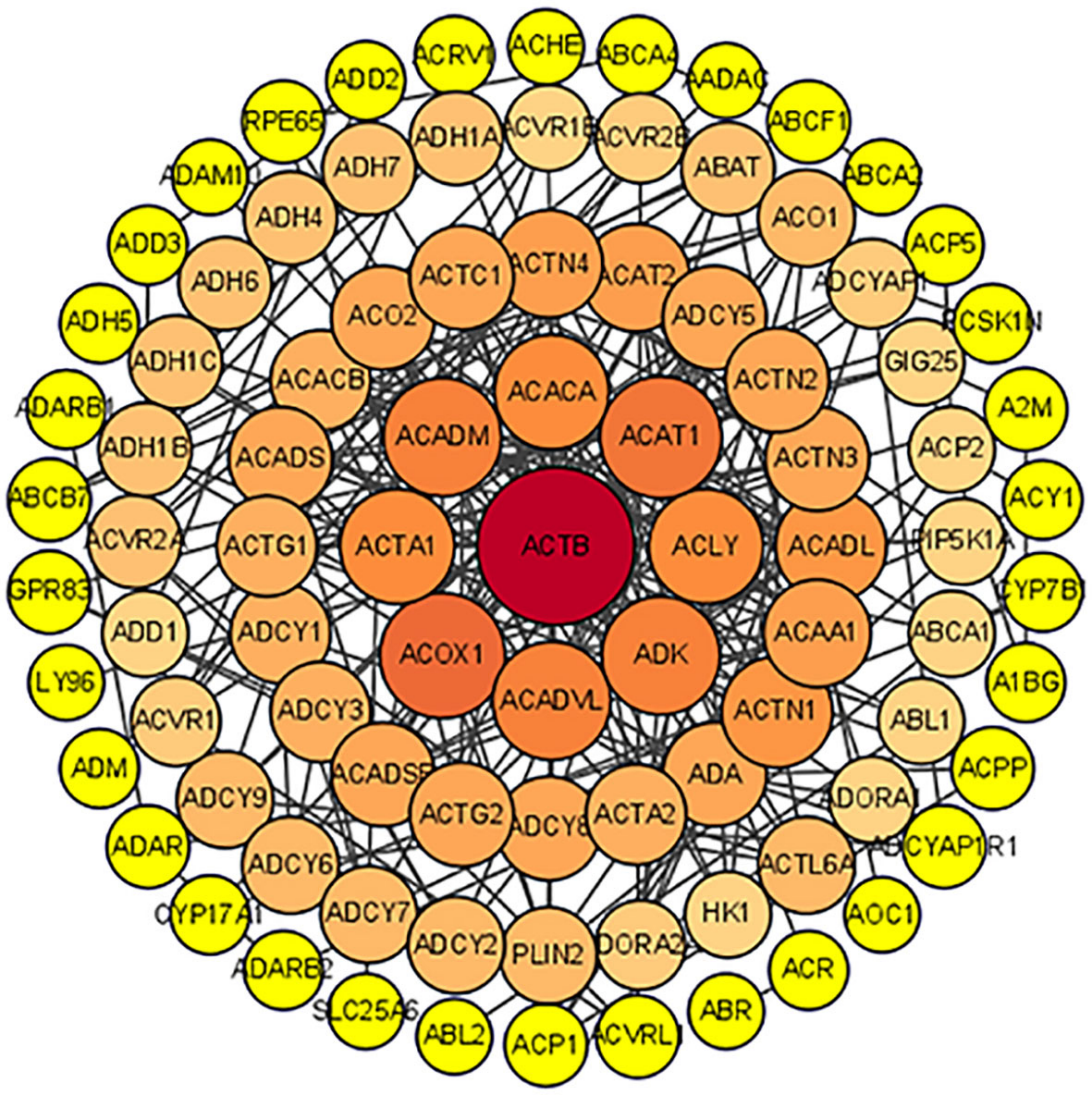
PPI network.

**Figure 4:**
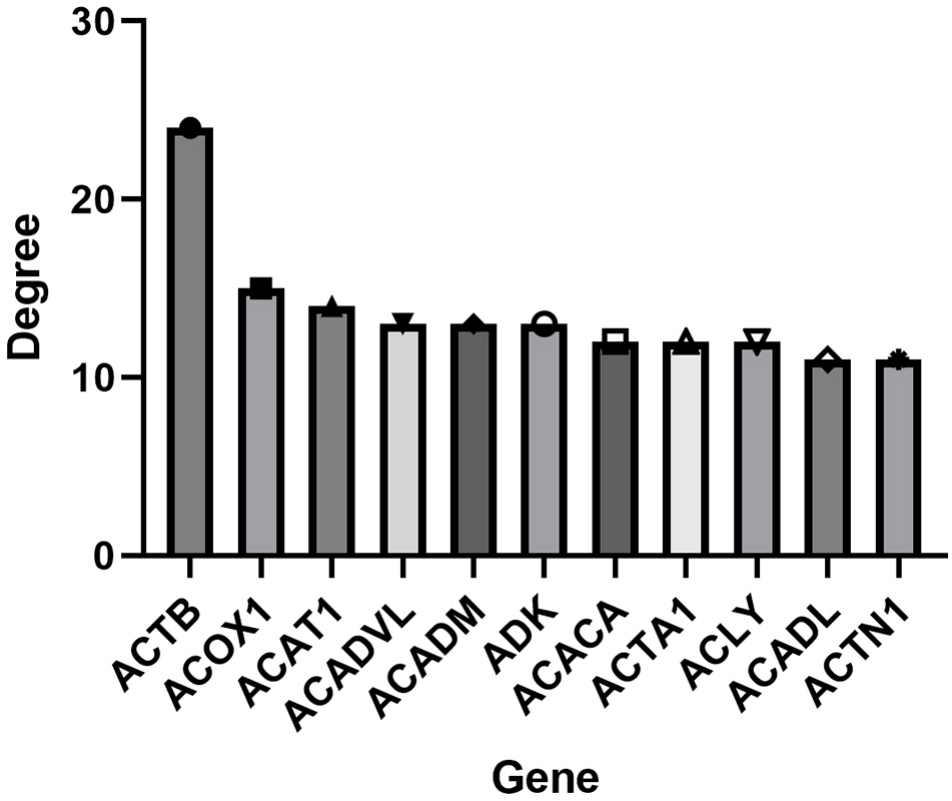
Top-11 hub genes of icaritin for UCEC treatment.

To further identify the genetic basis of the pharmacologic activities of icaritin, candidate genes were added to the STRING dataset to create a PPI network. The nodes and edges of the PPI network indicate proteins and protein–protein interactions. After omission of disconnected locations, a PPI network was formed with 91 notes and 266 edges (**Figure 3**). The top-11 key targets for potential anti-UCEC effects were actin-beta (ACTB), acyl-coenzyme a oxidase 1 (ACOX1), acetyl-coenzyme a acetyltransferase 1 (ACAT1), acyl coenzyme a dehydrogenase very long chain (ACADVL), acyl coenzyme A dehydrogenase (ACADM), adenosine kinase (ADK), actin-alpha 1 (ACTA1), adenosine triphosphate citrate lyase (ACLY), long-chain specific acyl-coa dehydrogenase (ACADL), and actinin alpha 1 (ACTN1) (**Figure 4**).

### Analysis of functional enrichment using the GO database

**Figure 5:**
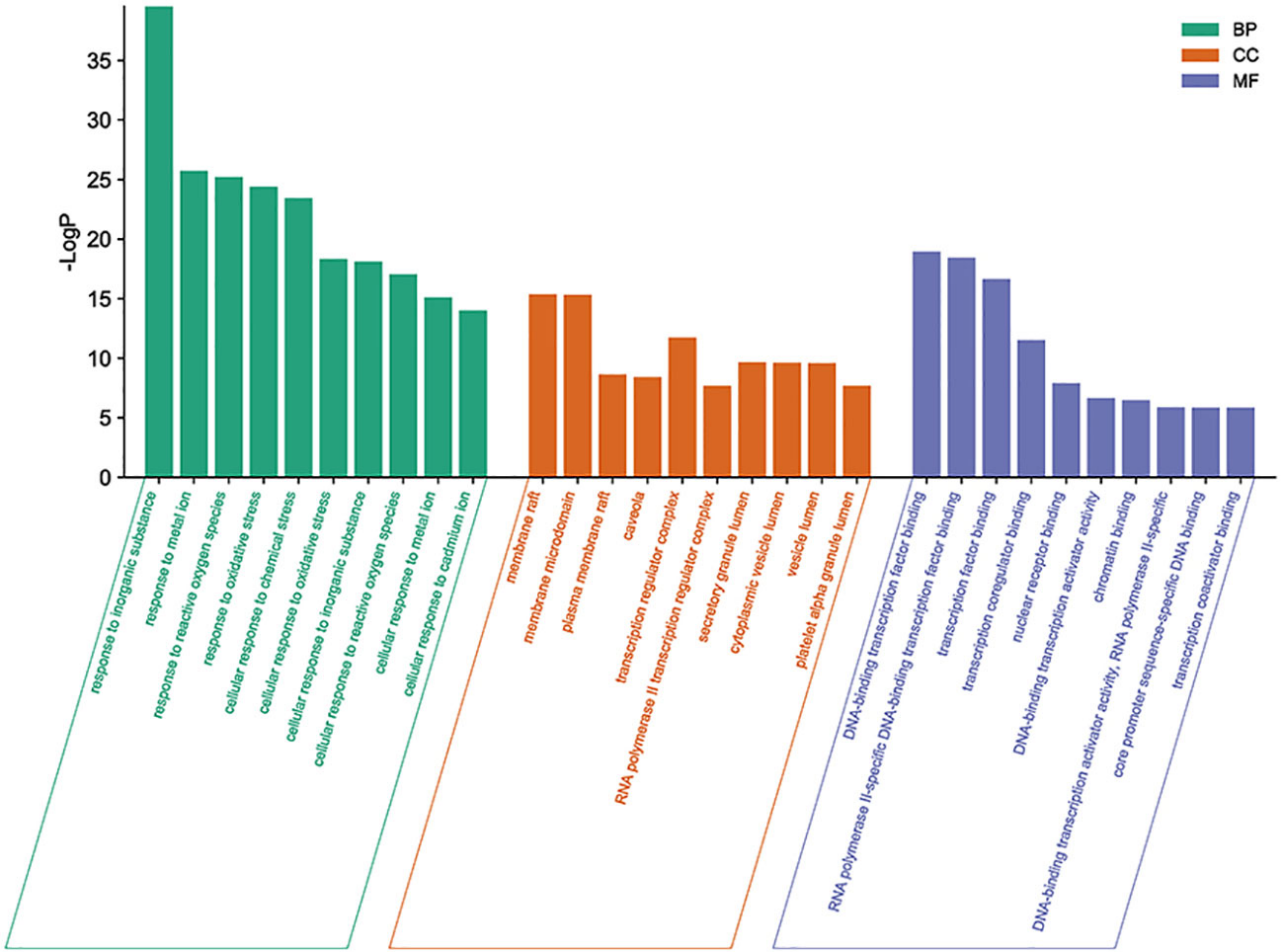
Top-10 enriched functions according to the GO database. The size of each dot denotes the variety of genes. The color of each dot correlates with the p-value.

The targets of icaritin stated above were inputted into the Metascape database to examine the vast diversity of biological properties of potential targets for UCEC therapy. The most enriched BP were “response to inorganic substance”, “response to metal ion”, and “response to reactive oxygen species.” The most enriched CC were “membrane raft”, “membrane microdomain”, and “plasma membrane raft”. The most enriched MF were “DNA-binding transcription factor binding”, “RNA polymerase II-specific DNA-binding transcription factor binding”, and “transcription factor binding” (**Figure 5**).

### Enrichment of signaling pathways according to the KEGG database

**Figure 6:**
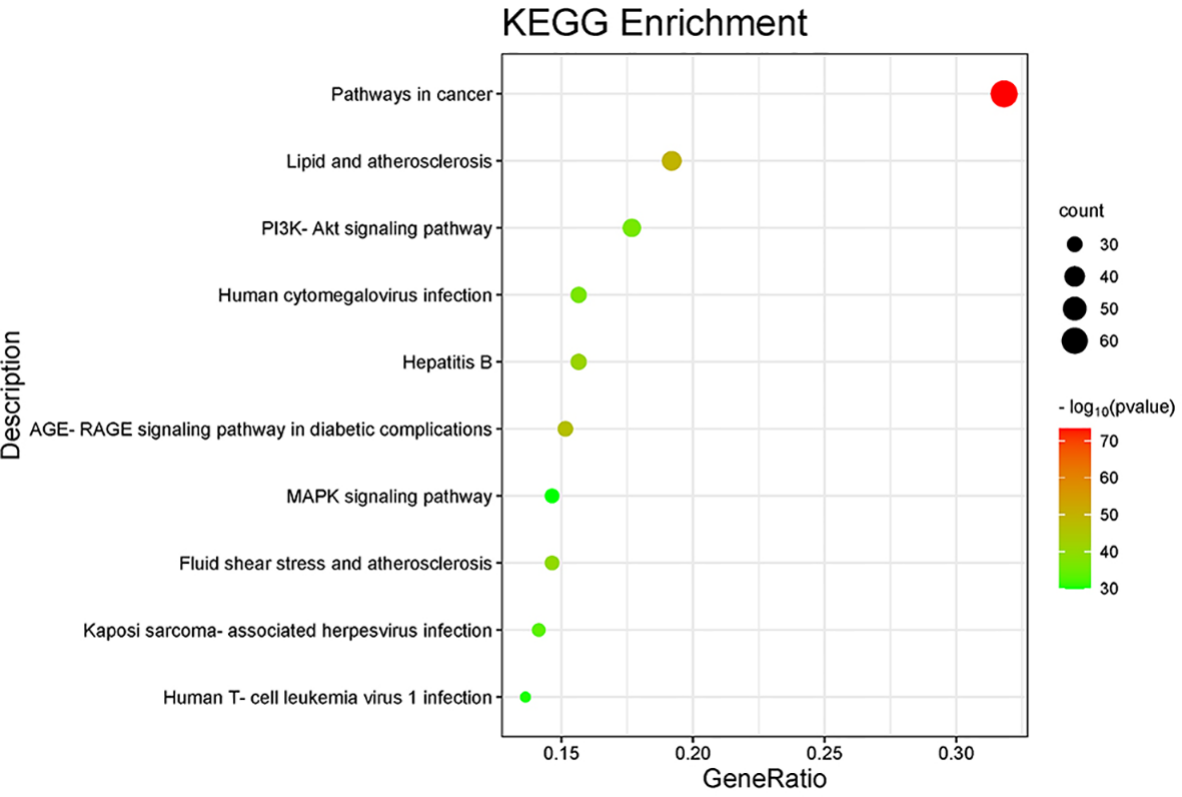
Enrichment of signaling pathways according to the KEGG database.

The 135 endpoints were entered into the Metascape database for analysis of enrichment of signaling pathways. A mixture of enrichment scores and p-values was used to determine the top-10 signaling pathways that were enriched (**Table 2**); “Pathways in cancer”, “PI3K-Akt signaling pathway”, and “MAPK signaling pathway” were among the signaling pathways enriched (**Figure 6**).

**Table 2:** Enriched signaling pathways in UCEC and their association with genes.

Pathway	Gene ratio	Count	Genes
Pathways in cancer	0.3181818	63	AKT1, BIRC5, AR, BAX, CCND1, BCL2, BCL2L1, CALM1, CASP3, CASP7, CASP8, CASP9, CCNA2, CDK2, CDK4,CDKN2A, E2F1, E2F2, EGF, ELK1, ERBB2, ESR2, FOS, GSK3B, GSTM1, GSTP1, HGF, HMOX1, HSP90AB1, IFNG, IGF2, IKBKB, IL2, IL4, IL6, CXCL8, MDM2, MMP1, MMP2, MMP9, MYC, NFE2L2, NFKBIA, NOS2, PPARD, PPARG, PRKCA, PRKCB, MAPK1, MAPK8, PTEN, PTGER3, PTGS2, RAF1, RELA, RXRA, RXRB, STAT1, TGFB1, TP53, VEGFA, NCOA1, RASSF1
Lipid and atherosclerosis	0.1919191	38	AKT1, BAX, BCL2, BCL2L1, CALM1, CASP3, CASP7, CASP8, CASP9, CD40LG, MAPK14, CYP1A1, FOS, GSK3B, HSPA5, HSP90AB1, ICAM1, IKBKB, IL1B, IL6, CXCL8, MMP1, MMP3, MMP9, NFE2L2, NFKBIA, NOS3, PPARG, PRKCA, MAPK1, MAPK8, RELA, RXRA, RXRB, SELE, TNF, TP53, VCAM1
PI3K-Akt signaling pathway	0.1767676	35	AKT1, CCND1, BCL2, BCL2L1, CASP9, CDK2, CDK4, COL1A1, EGF, ERBB2, ERBB3, GSK3B, HGF, HSP90AB1, IGF2, IKBKB, IL2, IL4, IL6, INSR, KDR, MCL1, MDM2, MYC, NOS3, PIK3CG, PRKCA, MAPK1, PTEN, RAF1, RELA, RXRA, SPP1, TP53, VEGFA
Hepatitis B	0.1565656	31	AKT1, BIRC5, BAX, BCL2, CASP3, CASP8, CASP9, CCNA2, CDK2, MAPK14, E2F1, E2F2, ELK1, FOS, IKBKB, IL6, CXCL8, MMP9, MYC, NFKBIA, PCNA, PRKCA, PRKCB, MAPK1, MAPK8, RAF1, RELA, STAT1, TGFB1, TNF, TP53
Human cytomegalovirus infection	0.1565656	31	AKT1, BAX, CCND1, CALM1, CASP3, CASP8, CASP9, CDK4, CDKN2A, MAPK14, E2F1, E2F2, ELK1, GSK3B, IKBKB, IL1B, IL6, CXCL8, MDM2, MYC, NFKBIA, PRKCA, PRKCB, MAPK1, PTGER3, PTGS2, RAF1, RELA, TNF, TP53, VEGFA
AGE-RAGE signaling pathway in diabetic complications	0.1515151	30	AKT1, BAX, CCND1, BCL2, CASP3, CDK4, COL1A1, COL3A1, MAPK14, F3, ICAM1, IL1A, IL1B, IL6, CXCL8, MMP2, NOS3, SERPINE1, PRKCA, PRKCB, MAPK1, MAPK8, RELA, SELE, STAT1, TGFB1, THBD, TNF, VCAM1, VEGFA
Fluid shear stress and atherosclerosis	0.1464646	29	AKT1, BCL2, CALM1, CAV1, MAPK14, FOS, GSTM1, GSTP1, HMOX1, HSP90AB1, ICAM1, IFNG, IKBKB, IL1A, IL1B, KDR, MMP2, MMP9, NFE2L2, NOS3, PLAT, MAPK8, RELA, SELE, THBD, TNF, TP53, VCAM1, VEGFA
MAPK signaling pathway	0.1464646	29	AKT1, CASP3, MAPK14, EGF, ELK1, ERBB2, ERBB3, FOS, HGF, HSPB1, IGF2, IKBKB, IL1A, IL1B, INSR, JUND, KDR, MYC, PRKCA, PRKCB, MAPK1, MAPK8, RAF1, RASA1, RELA, TGFB1, TNF, TP53, VEGFA
Kaposi sarcoma-associated herpesvirus infection	0.1414141	28	AKT1, BAX, CCND1, CALM1, CASP3, CASP8, CASP9, CDK4, MAPK14, E2F1, E2F2, FOS, GSK3B, ICAM1, IKBKB, IL6, CXCL8, MYC, NFKBIA, PIK3CG, MAPK1, MAPK8, PTGS2, RAF1, RELA, STAT1, TP53, VEGFA
Human T-cell leukemia virus-1 infection	0.1363636	27	AKT1, BAX, CCND1, BCL2L1, CCNA2, CDK2, CDK4, CDKN2A, CHEK1, E2F1, E2F2, ELK1, FOS, ICAM1, IKBKB, IL2, IL6, MYC, NFKBIA, MAPK1, MAPK8, PTEN, RELA, TGFB1, TNF, TP53,CHEK2

### Icaritin impairs the proliferation of HEC-1-A cells and enhances apoptosis

**Figure 7:**
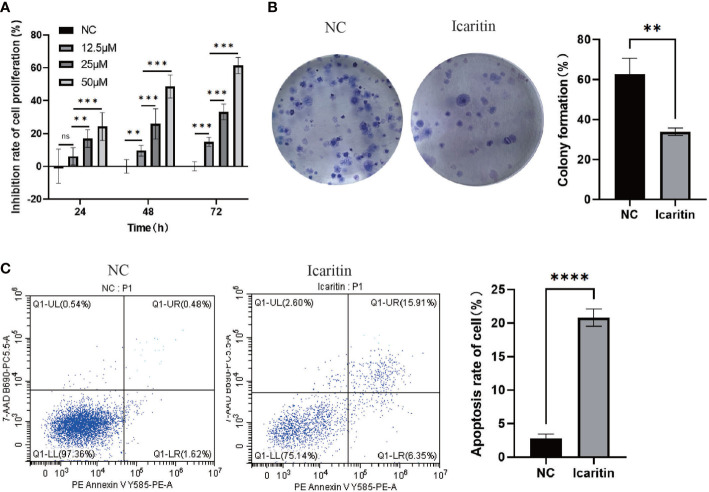
Icaritin impairs the proliferation, and enhances the apoptosis, of HEC-1-A cells. **(A)** Proliferation curves of HEC-1-A cells were created based on the CCK-8 assay 24h,48 h and 72h after addition of icaritin (12.5, 25, or 50 μM). **(B)** Colony-formation abilities of HEC-1-A cells were detected by Giemsa staining 2 weeks after addition of icaritin (25 μM). **(C)** Apoptosis of HEC-1-A cells was assessed 48 h after addition of icaritin (25 μM) by PE/annexin V/7AAD staining and flow cytometry. Images and quantitation are shown. Data are the mean ± SD, unpaired Student’s *t*-test, univariate analysis of variance was used to compare data between groups. **p<0.01, ***p<0.001, ns p>0.05, ****p<0.0001.

Addition of various concentrations of icaritin to HEC-1-A cells led to strong impairment of their viability (**Figure 7A**). Besides, the formation of colonies of HEC-1-A cells was also repressed when icaritin (25 μM) was added and incubation allowed to proceed for 48 h (**Figure 7B**). In addition, the number of apoptotic HEC-1-A cells increased after the addition of icaritin (25 μM) and incubation being allowed to proceed for 48 h (**Figure 7C**). These results indicated that icaritin could impede the growth and enhance the apoptosis of HEC-1-A cells.

### Icaritin impedes the migration of HEC-1-A cells

**Figure 8:**
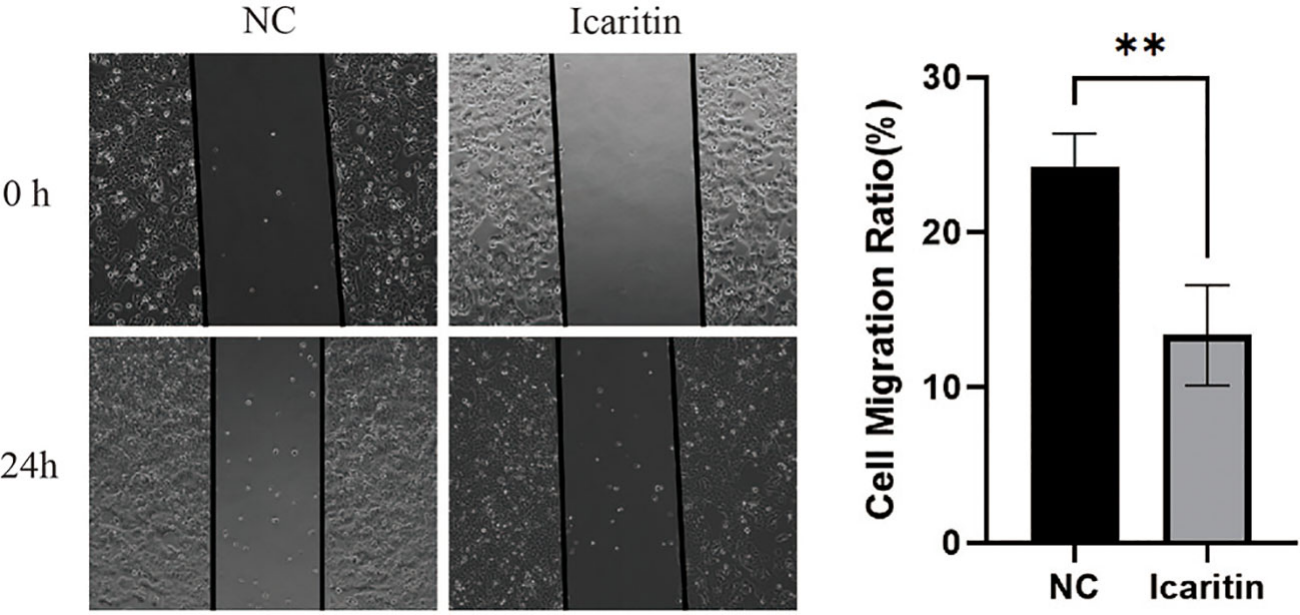
Icaritin impedes the migration of HEC-1-A cells. Migration capabilities of HEC-1-A cells 24 h after icaritin (25 μM) addition was evaluated by the wound-healing assay. Images and quantitation are shown. Data are the mean ± SD, unpaired Student’s *t*-test. **p<0.01.

To ascertain the biological function of icaritin in UCEC, we added medium containing icaritin (25 μM) to HEC-1-A cells and allowed incubation to proceed for 24 h. Icaritin inhibited the migration of HEC-1-A cells significantly according to the wound-healing assay (**Figure 8**). This result suggested that icaritin could impair the migration ability of HEC-1-A cells.

### Icaritin promotes cell proliferation by activating the PI3K/Akt pathway

**Figure 9:**
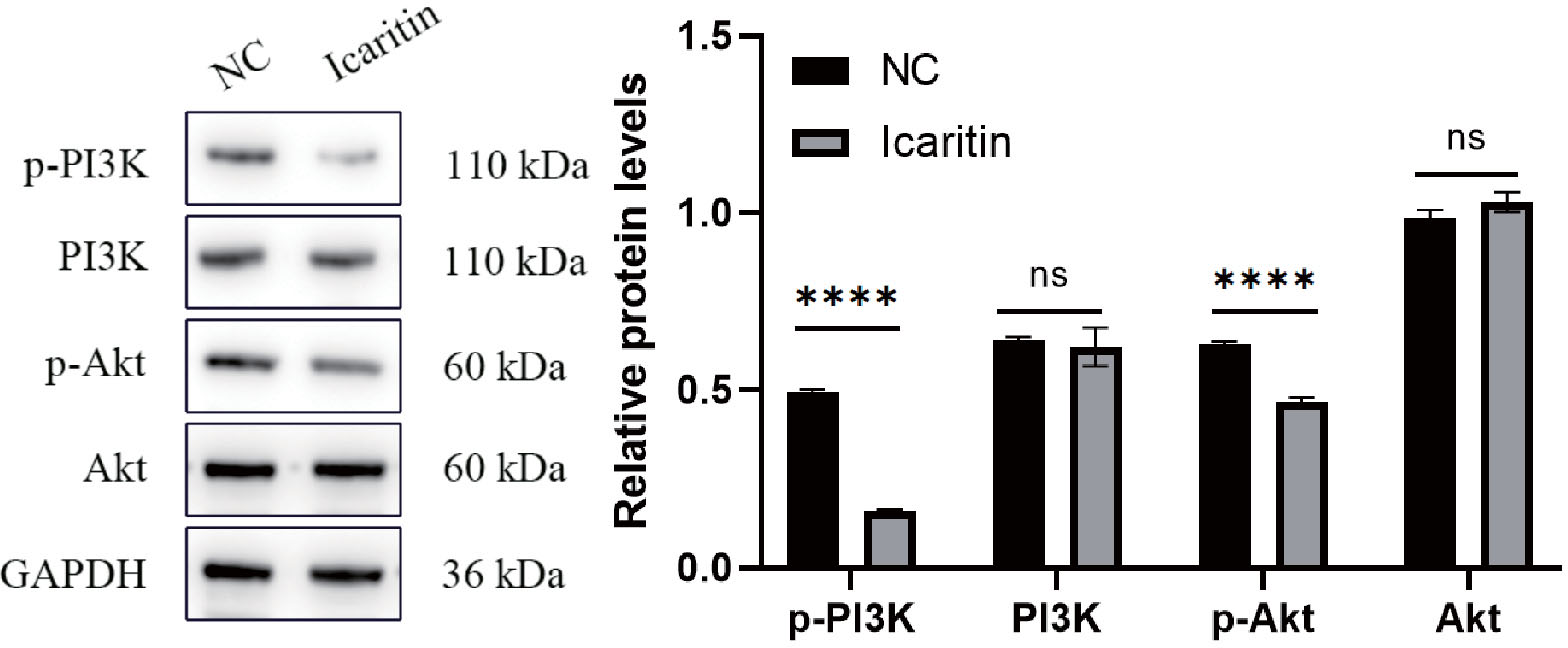
Compared with the NC group, the protein expression of phosphorylated phosphatidylinositol 3-kinase (p-PI3K) and phosphorylated protein kinase B (p-Akt) in the icaritin group was reduced significantly whereas, simultaneously, expression of PI3K and Akt was unchanged. Data are the mean ± SD, unpaired Student’s *t*-test. ****p<0.001; ns p>0.05.

Analyses of signaling-pathway enrichment using the KEGG database suggested that the PI3K-Akt signaling pathway was extremely important. The PI3K-Akt signaling pathway is important in tumor progression. To unravel the molecular mechanisms involved, we focused on this pathway. Interestingly, western blotting illustrated that when icaritin (25 μM) was added to HEC-1-A cells and incubation allowed to proceed for 48 h, expression of p-PI3K and p-Akt declined. Simultaneously, expression of PI3K and Akt was unchanged (**Figure 9**). These results illustrated that icaritin modulated activation of PI3K/Akt to promote the survival of HEC-1-A cells.

## Discussion

*Epimedium koreanum* and *Epimedium sagittatum* from the Berberidaceae family contain flavonoids such as icariin, epimedin A, and epimedin C (11). Icaritin has been used in TCM formulations for a long time, and some studies have been shown that it can inhibit tumor growth (12). The active components identified in our study, such as sitosterol, luteolin, and quercetin, have antitumor effects and inhibit tumors through various mechanisms. β-sitosterol has been shown to effectively reduce mitochondrial respiratory capacity by inhibiting mitochondrial complex I (13). Luteolin can reduce the size and weight of colonic tumors significantly by inhibiting cell-cycle arrest at the G2/M phase. This phenomenon involves inactivation of cyclin B1/cell-division cycle 2 and induction of apoptosis, in part *via* cytochrome c- and deoxyadenosine triphosphate-mediated activation of apoptotic protease activating factor-1 (14). Quercetin can suppress heterogeneous nuclear nucleoprotein A1-promoted apoptosis, reduce sphere-forming ability by cancer cells, and decrease cell proliferation (15).

We identified 135 candidate genes related to UCEC and icaritin. The top-11 key targets for potential anti-UCEC effects. Some studies have shown that ACOX1 may be involved in tumorigenesis. ACOX1 overexpression can mitigate, whereas downregulation of ACOX1 expression can promote, doxorubicin-induced apoptosis (16). Mitochondrial ACAT1 is the upstream acetyltransferase and deacetylase of pyruvate dehydrogenase alpha 1 (PDHA1) and pyruvate dehydrogenase phosphatase 1 (PDP1). Knockdown of ACAT1 expression attenuates tumor growth (17). ACADVL expression is downregulated, and ACADVL may be involved in, the pathogenesis of adrenocortical tumors (18). ACADM enhances the invasion and metastasis ability of breast cancer cells. Silencing of ACADM expression has been shown to suppress the migration and invasion of breast cancer cells significantly. In nude-mouse models, ACADM overexpression in MCF-7 cells has been shown to enhance their migration and invasion abilities significantly *in vivo* (19). Low ADK expression might be a risk factor and biomarker for cancer development. Use of an ADK inhibitor has been shown to inhibit kinetin riboside-induced apoptosis and cytotoxicity, which suggests that cancer-cell selectivity may be achieved based on ADK overexpression by cancer cells (20).

Functional-enrichment analysis using the GO database showed that the active components of icaritin had anti-tumor roles by participating in DNA transcription and oxidation reactions. Analyses of signaling-pathway enrichment using the KEGG database revealed that the PI3K-Akt, advanced glycation endproducts/receptor for advanced glycation endproducts (AGE-RAGE), mitogen-activated protein kinase (MAPK) and other signaling pathways were involved in UCEC. Hence, the active components of icaritin may play a part in multiple signaling pathways simultaneously. Studies have demonstrated activation of the PI3K-AKT signaling pathway in patients with UCEC, as well as its role in regulating the survival, growth, differentiation, and apoptosis of cancer cells (21). Accumulation of AGE is associated with upregulation of RAGE expression and activation of the AGE-RAGE axis, which leads to oxidative stress (22). The MAPK pathway has been revealed to be associated with UCEC progression (23). To further verify the exact target of icaritin and UCEC treatment, we conducted in-depth research on the PI3K-Akt signaling pathway.

Proliferation of HEC-1-A cells was detected using the CCK-8 assay. We found that icaritin inhibited the proliferation of HEC-1-A cells in a concentration-dependent manner. The effect of icaritin on apoptosis was detected by flow cytometry. Icaritin treatment could increase apoptosis, yet weaken the migration ability of cells.

Western blotting showed that protein expression of p-PI3K and p-Akt after icaritin treatment was reduced significantly yet, simultaneously, expression of PI3K and Akt was unchanged.

Hence, icaritin therapy could promote cell proliferation by activating the PI3K/Akt pathway and be used to treat UCEC. These data verified, once again, the reliability of network pharmacology to screen key genes and signaling pathways. Through the screening proffered by network pharmacology, we could identify many compounds from plants or TCM formulations to treat UCEC.

Our study had two main limitations. First, we investigated only at the cellular level in depth, which limited exploration of the pharmacologic effects of icaritin. Second, the data from Chinese herbal medicine-related Internet websites had limitations, such as a small sample size and lack of information on certain compounds (24).

## Conclusions

We demonstrated that icaritin has 23 bioactive components and 135 putative targets that are therapeutically important. The anti-tumor actions of ACOX1, ACAT1, ACADVL, and ACADM were identified as prospective targets. Icaritin had an anti-tumor role by participating in DNA transcription and oxidation reactions. PI3K-Akt and MAPK were the major signaling pathways involved in UCEC. Western blotting showed that expression of p-PI3K and Akt were reduced significantly after icaritin intervention. Hence, analyses of the active components of TCM formulations through network pharmacology may open-up new ideas for the treatment of diseases.

## Data availability statement

The datasets presented in this study can be found in online repositories. The names of the repository/repositories and accession number(s) can be found below: https://old.tcmsp-e.com/tcmspsearch.php?qr=Epimrdii%20Herba&qsr=herb_en_name&token=7fb472b8b3910154c805d916a6b60aae.

## Ethics statement

None of the employed cell lines required ethics approval for their use. Since no patients’ tissue or animals were examined, no statement regarding the ethics approval and consent to participate is needed.

## Author contributions

Y-BJ and SB conceived the research. Y-BJ and X-CL carried out the experiments. All authors participated in discussing and revising the manuscript. All authors approved the final version of the manuscript.
